# Improvement of Arterial Wall Lesions in Parallel with Decrease of Plasma Pentraxin-3 Levels in a Patient with Refractory Takayasu Arteritis after Treatment with Tocilizumab

**DOI:** 10.1155/2017/4580967

**Published:** 2017-06-06

**Authors:** Shiho Iwagaitsu, Taio Naniwa

**Affiliations:** ^1^Division of Rheumatology, Department of Internal Medicine, Nagoya City University Hospital, Nagoya, Japan; ^2^Department of Respiratory Medicine, Allergy and Clinical Immunology, Nagoya City University Graduate School of Medical Sciences, Nagoya, Japan

## Abstract

A 19-year-old Japanese woman with active Takayasu arteritis despite multiple conventional immunosuppressive therapies with glucocorticoids in combination with intravenous cyclophosphamide, azathioprine, or infliximab with methotrexate and tacrolimus was successfully treated by switching from infliximab to intravenous tocilizumab. Worsening of claudication of the legs and elevated acute phase reactants, including plasma pentraxin-3 levels, were observed during combination therapy with infliximab. Computed tomography demonstrated increased wall thickening with contrast enhancement in the preexisting lesion of the descending aorta and the femoral arteries. After switching from infliximab to tocilizumab, plasma pentraxin-3 levels gradually decreased to the normal range in parallel with the improvement of claudication. Follow-up computed tomographic scans confirmed the marked improvement of these arterial lesions. Moreover, plasma pentraxin-3 level was increased in response to the worsening of claudication that occurred just after switching to a subcutaneous tocilizumab injection. Measurements of plasma pentraxin-3 might be useful for evaluation of the vascular wall inflammation and therapeutic efficacy even during biologic therapy targeting tumor necrosis factor *α* and interleukin-6.

## 1. Introduction

Takayasu arteritis (TAK) is an idiopathic vasculitis mainly involving the aorta and its main branches. Glucocorticoids and conventional immunosuppressants are the mainstays of the treatment of TAK. Methotrexate and azathioprine are widely used, and cyclophosphamide is reserved for severe cases. Mycophenolate mofetil, cyclosporine, tacrolimus, and leflunomide have been tried in selected cases with successful results [1]. Proinflammatory cytokines, such as tumor necrosis factor- (TNF-) *α* and interleukin-6 (IL-6) have been shown to play a pivotal role in perpetuating disease activity of TAK [2–6], and biologics selectively targeting TNF-*α* and IL-6 signaling have been successfully used to treat refractory cases [7, 8].

Disease activity of large vessel vasculitis including TAK is usually estimated using clinical features, inflammatory markers, and imaging characteristics. As for inflammatory markers, C-reactive protein (CRP) and erythrocyte sedimentation rate (ESR), which reflect systemic inflammation, are commonly used to estimate disease activity of TAK. On the other hand, pentraxin-3, which belongs to a long pentraxin and is produced by various cells, such as macrophages, dendritic cells, fibroblasts, smooth muscle cells, adipocytes, vascular endothelial cells, and neutrophils, has been reported to correlate with vascular wall inflammation [9, 10]. Pentraxin-3 is induced by inflammatory cytokines, particularly by TNF-*α*, but not directly by IL-6. Thus, it can be assumed that anti-TNF therapy might mitigate the response of pentraxin-3 to vascular wall inflammation, as observed in CRP and ESR levels during anti-IL-6 therapy.

Here, we report on a patient with refractory TAK despite treatment with infliximab, who was successfully treated with tocilizumab. The effects on the arterial lesions were evaluated by computed tomography as well as of inflammatory markers including plasma pentraxin-3. Written informed consent was obtained from the patient for publication of this case report and accompanying images.

## 2. Case Presentation

**Figure 1:**
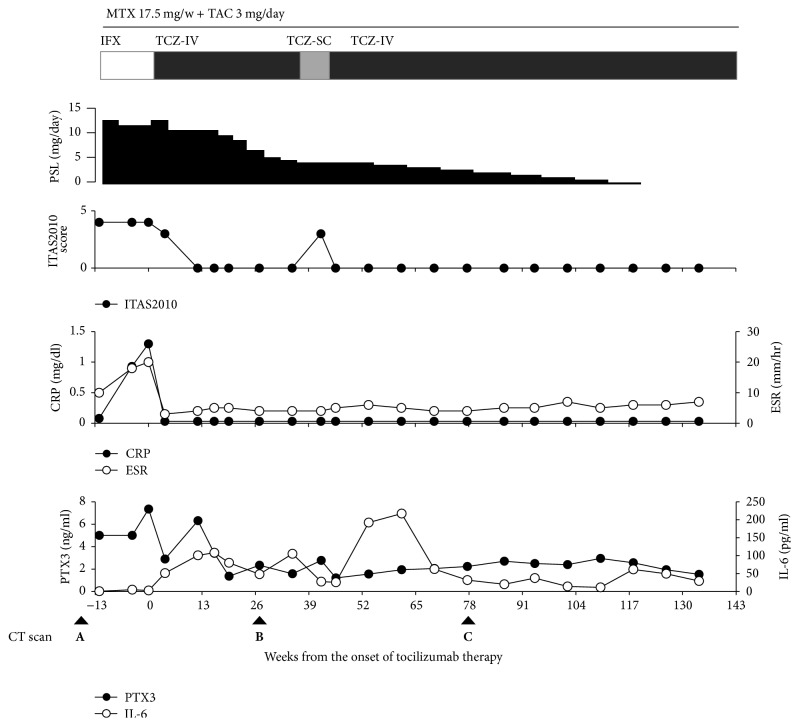
Changes in disease activity, biomarkers, and treatment during anticytokine biologic therapy. Infliximab (IFX) was administered at a dose of 6 mg per kilogram of body weight every four weeks (white bar). Intravenous tocilizumab (TCZ-IV) was administered at a dose of 8 mg per kilogram of body weight every four weeks (black bar), and subcutaneous tocilizumab (TCZ-SC) was administered at a dose of 162 mg per body weight every other week (gray bar). CRP, C-reactive protein; ESR, erythrocyte sedimentation rate; IL-6, interleukin-6; ITAS2010, The Indian Takayasu Activity Score 2010; PSL, prednisolone; PTX3, pentraxin-3.

**Figure 2:**
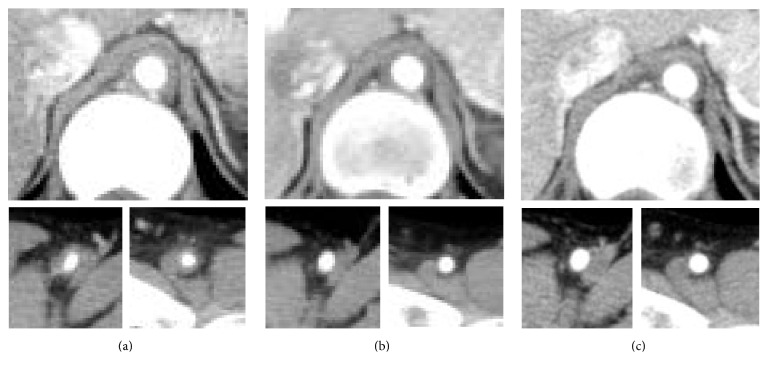
Changes in the computed tomographic findings of arterial lesions of Takayasu arteritis before and after starting tocilizumab therapy. Contrast-enhanced CT of the descending aorta (the upper), the right femoral artery (the bottom left), and the left femoral artery (the bottom right) was performed. Three months before starting tocilizumab therapy, while on the treatment with infliximab, arterial wall thickening of the femoral arteries was observed (a). After six months of tocilizumab therapy, there was a significant decrease in the wall thickness of the descending aorta and the femoral arteries (b). After 17 months of tocilizumab therapy, there was a further decrease in arterial wall thickness and an increase in the intraluminal diameter of the descending aorta and the femoral arteries (c).

A 19-year-old Japanese woman with active TAK refractory to combination therapy with conventional immunosuppressants and infliximab was given tocilizumab therapy. She was diagnosed as having TAK at age 15 when she developed abdominal pain, right carotidynia, low-grade fever, and claudication in both legs and the left arm. Contrast-enhanced computed tomography (CT) revealed wall thickening and stenosis of the descending aorta near of the aortic hiatus and main branches of the aorta. The thickened wall of the affected portion of the aorta was enhanced by contrast material. CRP and ESR were elevated at 6.03 mg/dl and 122 mm/hour, respectively. She was initially treated with 40 mg per day of prednisolone. She developed reversible cerebral vasoconstriction syndrome in the initial treatment course of TAK, which was described in our previous report on this patient [11]. She relapsed on prednisolone 15 mg per day, was given azathioprine with no remarkable response, and required dose increments of prednisolone up to 40 mg per day. Following that, therapy with intravenous cyclophosphamide and methotrexate was instituted but failed to control her disease activity. One year before starting tocilizumab, she started infliximab, whose dose was increased to 6 mg per kilogram of body weight, every four weeks after week 10, in addition to prednisolone 6 mg per day and methotrexate 17.5 mg per week. Six months later, CT showed exacerbation of the preexisting lesion of the descending aorta and newly emerged lesions in both femoral arteries. Then prednisolone dose was increased to 20 mg per day, and tacrolimus 3 mg per day was added. Three months before starting tocilizumab, she again developed claudication in both legs and the left arm (Figure 1). CRP and ESR levels had been 0.08 to 1.3 mg/dl and 10 to 20 mm/hour, respectively. Plasma pentraxin-3 levels measured using the Human Pentraxin3/TSG-14 ELISA System, by Perseus Proteomics Inc. Tokyo, Japan, had remained high ranging from 5.00 to 7.35 ng/ml during the last three months of infliximab therapy. The geometric mean [confidence intervals] of plasma pentraxin-3 levels in healthy Japanese woman was reported to be 2.12 [2.05, 2.19] ng/ml [12]. We switched from infliximab to tocilizumab, 8 mg per kilogram of body weight, every four weeks. Improvement of claudication of symptoms was observed in four weeks and continued over six months. Contrary to CRP and ESR, which were decreased to <0.03 mg/dl and 3 mm/hour at four weeks, pentraxin-3 levels had decreased with fluctuation over five months after starting tocilizumab. By her wish, we switched from intravenous tocilizumab to subcutaneous form, 162 mg per body, every other week, at 35 weeks, and when pentraxin-3 levels were 1.59 ng/ml. Four weeks later, claudication in the left arm was worsened when we switched the form of tocilizumab back to intravenous form. CRP and ESR had remained unchanged, but pentraxin-3 levels were slightly increased to 2.77 ng/ml. Four weeks after resuming intravenous tocilizumab, pentraxin-3 levels were decreased to 1.22 ng/ml. Just after switching to subcutaneous tocilizumab, the transient decrease of serum IL-6 and subsequent increase of IL-6 were observed. CT obtained one year after starting tocilizumab revealed normalization of the appearance of both femoral arteries and improvement of the arterial wall thickening in the lesion of the descending aorta (Figure 2). Tocilizumab treatment could allow tapering off prednisolone over 27 months. No serious adverse events were observed during tocilizumab treatment.

## 3. Discussion

Previous reports have shown that anti-TNF and anti-IL-6 therapies were equally efficacious in patients with active TAK refractory to conventional therapies [7]. Although there are limited data on the efficacy of tocilizumab in patients with active TAK, despite anti-TNF therapy, the efficacy of tocilizumab was observed in more than half of the reported cases of refractory to anti-TNF-*α* therapy [13, 14]. In the present case, marked improvement in the clinical and radiographic signs of TAK was observed after switching from infliximab to tocilizumab.

We measured serial plasma pentraxin-3 levels, which are reported to correlate with vascular wall inflammation of TAK [15], as well as CRP and ESR during infliximab and tocilizumab therapy. During the last three months of infliximab therapy, which was clinically and radiographically ineffective, serum CRP and ESR levels remained normal to slightly positive, 0.08 to 1.3 mg/dl and 10 to 20 mm/hour, respectively. Although pentraxin-3 is induced by inflammatory cytokines, particularly by TNF-*α*, plasma pentraxin-3 levels remained high, 5.00 to 7.35 ng/ml in the presence of active vascular wall lesions despite treatment with anti-TNF therapy. These findings suggest that plasma pentraxin-3 could reflect vascular wall inflammation during anti-TNF therapy.

Tocilizumab, an IL-6 receptor antagonist, directly inhibits the production of downstream acute phase reactants, including CRP, and it may normalize CRP and ESR, irrespective of the disease activity of inflammatory diseases. Thus, these classic markers might be of limited value as surrogate markers for estimating disease activity in TAK in patients receiving tocilizumab. In the present case, CRP and ESR levels promptly decreased to and remained in undetectable quantities and near the minimum of the normal range, respectively, after starting the tocilizumab therapy. On the other hand, plasma pentraxin-3 had decreased with the fluctuations but normalized within five months of starting tocilizumab. A marked improvement of claudication was observed during the first four weeks of the tocilizumab treatment, while further improvements of claudication continued for the following six months. Furthermore, plasma pentraxin-3 levels increased during a symptomatic flare, which increased the Indian Takayasu Activity Score (ITAS2010) [16], directly after temporarily switching to a subcutaneous tocilizumab injection. At the same time, a temporary decrease in serum IL-6 levels and a subsequent increase in IL-6 levels were observed after resuming intravenous tocilizumab infusion. Serum IL-6 levels in the course of tocilizumab therapy with sufficient doses will reflect the actual endogenous IL-6 production that correlates with the true disease activity, while inflammatory symptoms are ameliorated by the inhibition of IL-6 signaling through IL-6 receptors [17]. It can be assumed that temporal change of IL-6 levels after switching to subcutaneous tocilizumab might be due to decreased saturated ratio of IL-6 receptor with tocilizumab caused by lower blood concentration of tocilizumab, which made room for free IL-6 to bind to IL-6 receptor and might cause less sufficient inhibition of IL-6 signaling that contributes to worsening of disease activity of TAK.

Previous literature has shown that plasma pentraxin-3 levels were similar in patients with active or inactive disease defined by clinical disease activity measures, such as National Institute of Health criteria and ITAS2010 [18]. On the other hand, plasma pentraxin-3 levels were increased in the presence of vascular wall enhancement by contrast-enhanced magnetic resonance imaging and ^18^F-Fluorodeoxyglucose Uptake by Positron Emission Tomography, which could reflect ongoing arterial wall inflammation [19]. In the present case, plasma pentraxin-3 levels seemed to correlate well with clinical manifestation related to active arterial lesions, which were confirmed by serial assessments with CT. Thus, plasma pentraxin-3 might be a useful biomarker for evaluation of the vascular wall inflammation and therapeutic efficacy even in patients receiving anti-TNF and anti-IL-6 biologic therapy, especially in those receiving the latter, which could normalize CRP and ESR irrespective of inflammatory disease activity.

In summary, our most important observation from the present case is that serial change of plasma pentraxin-3 levels could reflect vascular wall inflammation and the core pathology of TAK under treatment with these anticytokine biologics. This observation implicates the need for further exploration of plasma pentraxin-3 as a biomarker for estimating disease activity of TAK under treatment with these anticytokine biologics in a larger clinical study. Furthermore, the remission-inducing efficacy of tocilizumab in this patient with active TAK despite treatment with anti-TNF biologics highlights a crucial role of interleukin-6 in perpetuating disease activity of TAK.
